# Risk factors for suboptimal visual outcomes after cataract surgery: A retrospective study in Guyana

**DOI:** 10.1097/MD.0000000000045163

**Published:** 2025-10-17

**Authors:** Yi Liu, Shailendra Sugrim, Shivannie Persaud, Carla Reid, Tiffony Persaud, Caroline Mingo

**Affiliations:** a Department of Ophthalmology, Affiliated Hospital of Jiangnan University, Wuxi, China; b Georgetown Public Hospital Corporation, Georgetown, Guyana.

**Keywords:** cataract surgery, developing countries, ocular comorbidities, visual acuity, zonular laxity

## Abstract

This study aims to assess visual outcomes following cataract surgery and identify key risk factors contributing to suboptimal postoperative vision in Guyana. This retrospective study included 103 patients who underwent cataract surgery between December 2023 and June 2024. Preoperative and postoperative visual acuity were recorded, and patients were categorized into low vision or normal vision groups based on best-corrected visual acuity (BCVA) at 1 month post-surgery. Logistic regression analysis was conducted to identify factors associated with poor visual outcomes. One month after surgery, 24 patients were classified into the low vision group, while 79 had normal vision according to their BCVA. Preoperatively, 96% of patients in the low vision group had BCVA of 20/200 or worse, significantly higher than the 60.8% observed in the normal vision group (*P* < .05). Ocular comorbidities were present in 67% of patients in the low vision group, significantly higher than the 12.7% observed in the normal vision group (*P* < .05). Additionally, zonular abnormalities or posterior capsule rupture were found in 33% of the low vision group, compared to only 5% in the normal vision group (*P* < .05). No statistically significant differences were observed between the 2 groups concerning age, gender, small pupil presence, iris relaxation, or systemic comorbidities. Multivariate logistic regression identified ocular comorbidities and intraoperative zonular abnormalities or posterior capsule rupture as independent risk factors for poor postoperative vision (*P* < .05). Coexisting ocular comorbidities and intraoperative zonular laxity or posterior capsule rupture are significant risk factors for suboptimal postoperative vision in resource-limited settings like Guyana. Optimizing preoperative assessment and implementing tailored postoperative care strategies may enhance outcomes for high-risk patients.

## 1. Introduction

Cataract remains the leading cause of preventable blindness worldwide, particularly in developing countries where access to surgical interventions is limited. Despite significant advancements in cataract surgery in high-income countries, the outcomes and accessibility of these surgeries remain a challenge in resource-constrained regions like Guyana.^[1–3]^ Guyana, located near the equator, experiences high ultraviolet exposure, which contributes to a high prevalence of cataracts. However, the surgical infrastructure and resources required for high-quality outcomes are often lacking, leading to varied postoperative results.

To date, no previous studies have examined the postoperative visual outcomes of cataract surgery in Guyana, making this the first investigation into the unique challenges and successes of cataract surgery in this region. Insights from this study are essential for developing targeted strategies to improve surgical outcomes and address the specific needs of patients in similar resource-limited settings.^[4]^

This study aims to assess postoperative visual outcomes and identify risk factors for suboptimal vision in cataract patients in resource-limited Guyana. By highlighting the factors associated with poor postoperative vision, this research aims to inform better surgical planning and postoperative care practices, ultimately improving patient outcomes in challenging environments. The risk factors examined in this study included demographic characteristics, systemic comorbidities, ocular comorbidities, and intraoperative complications.

## 2. Methods

### 2.1. Patients selection

This retrospective study analyzed records of 103 patients diagnosed with senile cataracts who underwent cataract surgery at the Georgetown Public Hospital Corporation (GPHC) in Guyana from December 2023 to June 2024. Cases were eligible for inclusion if they had been diagnosed with senile cataracts at the clinic and were aged ≥50 years.They were excluded if they were unable to communicate (e.g., due to dementia, deafness), or it was not recommended they undergo cataract surgery (e.g., serious medical illness or ophthalmic disease other than cataract thought to be the main cause of blindness). Only 1 eye from each patient was included in the study. When both eyes underwent surgery, the eye with poorer preoperative visual acuity was selected for analysis to avoid inter-eye correlation. Routine preoperative examinations included uncorrected and best-corrected visual acuity (BCVA), intraocular pressure measurement, slit-lamp examination, dilated lens and fundus examination, and corneal curvature and axial length measurements using A/B-scan ultrasound, due to limited access to optical biometry. Blood glucose and blood pressure measurements were obtained for patients with systemic comorbidities like diabetes and hypertension, although comprehensive systemic control was often limited by local resource constraints. This retrospective study was approved by the Ethics Committee of GPHC, Guyana. The study was conducted in accordance with the principles of the Declaration of Helsinki. The clinical data were accessed between December 2023 and June 2024 for research purposes. All patient data were anonymized prior to analysis, and the authors did not have access to any personally identifiable information. This study is retrospective in nature, and the requirement for informed consent was waived by the Ethics Committee of GPHC, Guyana.

### 2.2. Treatment

Most patients underwent standard phacoemulsification with intraocular lens implantation. In cases with significant zonular laxity or posterior capsule rupture, conversion to small incision cataract surgery or extracapsular cataract extraction was performed, as capsular tension rings were unavailable. Intraoperative complications, such as posterior capsular rupture and zonular instability, were documented.

### 2.3. Follow-up

Postoperative care included a 1-month regimen of corticosteroid and antibiotic eye drops. Patients were followed up on the first day, 1 week, and 1 month postoperatively to assess visual acuity and monitor for complications.

### 2.4. Statistical analysis

Data analysis was conducted using SPSS 22.0. Continuous variables were analyzed using *t* tests, and categorical variables were assessed with chi-square tests. Logistic regression model was performed to identify independent risk factors for poor visual outcomes, with statistical significance set at *P* < .05.

## 3. Results

### 3.1. Baseline characteristics of participants

Between December 2023 to June 2024, a total of 103 cataract surgeries were performed. One month after surgery, 24 patients were classified as having low vision, while 79 were classified as having normal vision based on their BCVA.

**Figure 1.**
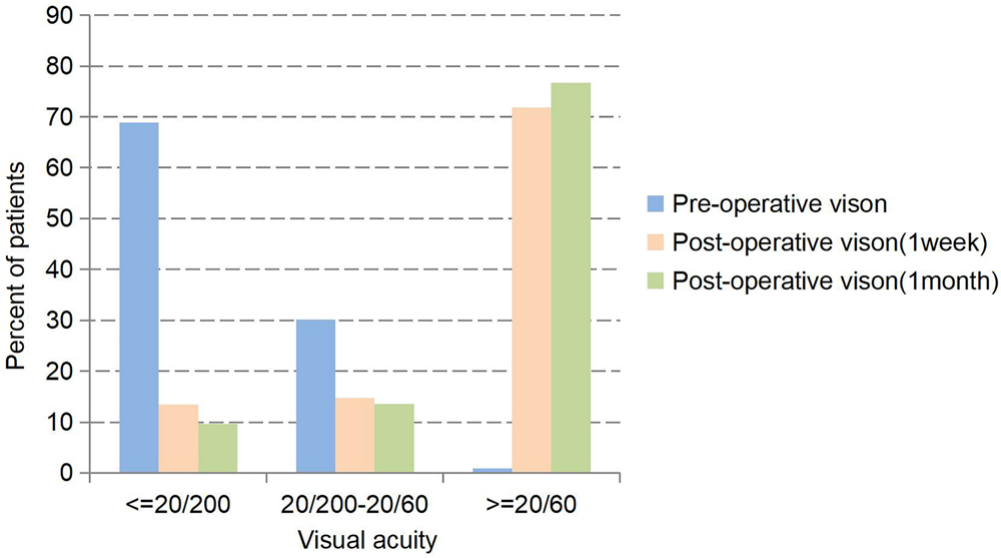
Comparison of pre- and postoperative visual acuity in cataract surgery patients.

Table 1 shows the baseline characteristics of 103 patients enrolled in this study. Among the 103 elderly cataract patients, the average age was 67.86 ± 9.32 years. There were 44 male and 59 female patients. A total of 68 patients had a history of systemic comorbidities, such as hypertension, diabetes, or ankylosing spondylitis. Twenty-six patients had coexisting ocular conditions, including glaucoma, uveitis, or diabetic retinopathy and so on (some patients had more than 1 systemic or ocular comorbidity). Intraoperatively, 29 patients were found to have pupil constriction or iris relaxation, and 12 patients had zonular laxity or posterior capsule rupture. Figure 1 shows the pre- and postoperative visual acuity in the operated eye. Among the patients, 71 (68.9%) had preoperative visual acuity of 20/200 or worse, 31 (30.2%) had visual acuity better than 20/200 but <20/60, and only 1 patient (0.9%) had visual acuity of 20/60 or better. One week after operation, 74 patients (71.8%) had visual acuity of 20/60 or better, 52 patients (50.5%) had visual acuity of 20/40 or better, and 1 month after surgery, 79 patients (76.7%) had visual acuity of 20/60 or better, 73 patients (70.9%) had visual acuity of 20/40 or better.

**Table 1:** Baseline characteristics of cataract surgery patients.

Variables	Number of patients (%)
Age (yr)	
≥50, ≤59	19 (18.4)
≥60, ≤69	30 (29.1)
≥70	52 (50.4)
Eye	
Right eye	62 (60.1)
Left eye	41 (39.8)
Gender	
Male	44 (42.7)
Female	59 (57.2)
Preoperative BCVA	
<20/200	71 (68.9)
≥20/200, ≤20/60	32 (31.0)
Systemic comorbidities	
Diabetes	35 (33.9)
Hypertension	50 (48.5)
HIV	1 (0.9)
AS	1 (0.9)
Heart diseases	4 (3.8)
Respiratory disease	4 (3.8)
Ocular comorbidities	
Glaucoma	16 (15.5)
DR	7 (6.7)
Macular diseases	4 (3.8)
Uveitis	1 (0.9)
PEX	1 (0.9)

AS = ankylosing spondylitis, BCVA = best-corrected visual acuity, DR = diabetic retinopathy, PEX = pseudoexfoliation syndrome.

### 3.2. Complications

**Figure 2.**
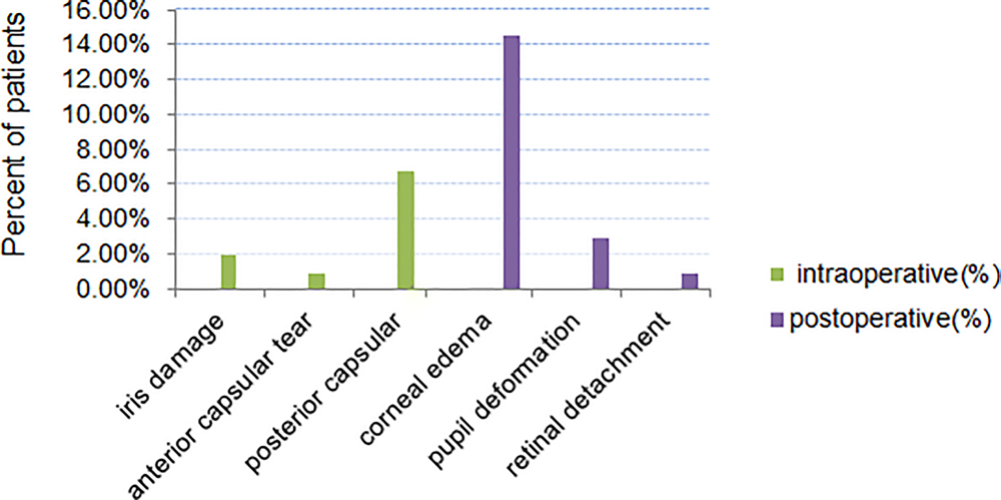
Incidence of intraoperative and postoperative complications.

Among the 103 patients, 2 underwent intracapsular cataract extraction due to extensive zonular dehiscence, and 1 patient switched to small incision cataract surgery due to a hard nucleus. The remaining 100 patients all underwent phacoemulsification. Figure 2 shows intraoperative and postoperative complications of 103 patients, Intraoperative complications mainly included iris damage in 2 cases (1.9%), posterior capsular rupture in 7 cases (6.7%), and anterior capsular tear in 1 case (0.9%). Postoperative complications primarily consisted of corneal edema in 15 cases (14.5%), most of which were mild, manifesting as stromal edema near the main incision. Patients with a nucleus hardness of grade IV or higher also presented with central corneal stromal edema accompanied by Descemet membrane folds. All patients’ corneal edema had resolved by the 1-month postoperative follow-up after treatment with topical dexamethasone eye drops. Pupil deformation occurred in 3 cases (2.9%), primarily due to iris injury caused by small pupils during surgery. Retinal detachment occurred in 1 case (0.9%), and this patient had a history of previous ocular trauma. No serious complications such as corneal decompensation, lens nucleus drop into the vitreous cavity, or endophthalmitis were observed in this group of patients.

### 3.3. Differences in characteristics between low and normal vision groups

Significant differences were found in preoperative visual acuity, ocular comorbidities, and the incidence of zonular abnormalities or posterior capsular rupture between the 2 groups. Based on the BCVA 1 month after operation, 24 patients were classified into the low vision group (BCVA <20/60), and 79 patients into the normal vision group (BCVA 20/60 or better). Table 2 shows the comparison of the factors affecting vision between the 2 groups of patients. In the low vision group, the proportion of patients with preoperative vision of 20/200 or worse was 96%, while in the normal vision group, this proportion was only 60.8% (*P* < .05). The proportion of patients with ocular comorbidities in the low vision group was 67%, compared to only 12.7% in the normal vision group (*P* < .05). The proportion of patients with zonular abnormalities or posterior capsule rupture in the low vision group was 33%, compared to only 5% in the normal vision group (*P* < .05). Comparisons of age, gender ratio, the presence of small pupils and iris relaxation, and systemic comorbidities between the 2 groups showed no significant differences.

**Table 2:** Comparison of factors influencing postoperative visual acuity between low vision and normal vision group.

Influencing factors	Low vision group	Normal vision group	*P*-value
	n = 24	n = 79	
Gender			.913
Male	10 (41.7%)	34 (43.0%)	
Female	14 (58.3%)	45 (57.0%)	
Age (years)			.272
≥50, ≤59	3 (12.5%)	15 (19.0%)	
≥60, ≤69	4 (16.7%)	26 (33.0%)	
≥70	17 (70.8%)	38 (48.0%)	
Preoperative visual acuity			<.001
≤20/200	23 (95.8%)	48 (60.8%)	
>20/200	1 (4.2%)	31 (39.2%)	
Systemic medical history			.931
Yes	16 (67.0%)	52 (66.0%)	
No	8 (33.0%)	27 (34.0%)	
Ocular comorbidities			<.001
Present	16 (67%)	10 (12.7%)	
Absent	8 (33%)	69 (87.3%)	
Small pupils and iris relaxation			.901
Present	7 (29.1%)	22 (27.8%)	
Absent	17 (70.8%)	57 (72.2%)	
zonular laxity or posterior capsule rupture (Y/N)			<.001
Present	8 (33.3%)	4 (5.0%)	
Absent	16 (66.7%)	75 (95.0%)	

### 3.4. Univariable and multivariate logistic regression analysis outcomes

The results of univariable shows that ocular comorbidities (odds ratio [OR] = 8.55, 95% confidence interval [CI]: 6.04–56.97, *P* < .001) and intraoperative zonular laxity or posterior capsular rupture(OR = 9.37, 95% CI: 2.51–34.95, *P* < .001) are important factors for low vision following cataract surgery (Table 3). Multivariate logistic regression analysis further indicates that ocular comorbidities (OR = 9.13, 95% CI: 6.90–64.94, *P* < .001) and intraoperative zonular laxity or posterior capsular rupture (OR = 9.23, 95% CI: 3.43–39.74, *P* < .001) are independent risk factors for low vision following cataract surgery (Table 3). Other variables, including age, gender, and systemic comorbidities, did not reach statistical significance.

**Table 3:** Univariable and multivariate logistic regression analysis of factors influencing postoperative low vision.

Characteristic	Univariable		Multivariable	
	OR (95% CI)	*P*	OR (95% CI)	*P*
Age	1.03 (0.98–1.09)	.272	1.10 (1.00–1.22)	.058
Gender				
Male	Ref		Ref	
Female	1.06 (0.42–2.67)	.905	0.15 (0.01–2.37)	.177
Systemic comorbidities				
No	Ref		Ref	
Yes	0.87 (0.34–2.23)	.765	0.19 (0.02–2.36)	.198
ocular comorbidities				
No	Ref		Ref	
Yes	8.55 (6.04–56.97)	<.001	9.13 (6.90–64.94)	<.001
Small pupils and iris relaxation				
No	Ref		Ref	
Yes	0.81 (0.29–2.30)	.695	2.72 (0.29–25.17)	.377
Zonular laxity or posterior capsule rupture				
No	Ref		Ref	
Yes	9.37 (2.51–34.95)	<.001	9.23 (3.43–39.74)	<.001
Preoperative visual acuity	0.73 (0.38–2.43)	.998	0.69 (0.12–3.26)	.999

CI = confidence interval, OR = odds ratio.

## 4. Discussion

Visual outcomes after cataract surgery vary considerably across geographic regions, reflecting differences in patient populations, healthcare infrastructure, and surgical techniques. In our study, 9.7% of eyes failed to achieve a BCVA of ≥6/60 1 month postoperatively, falling short of the World Health Organization benchmark of <5% poor outcomes.^[5]^ While this figure is better than those reported in some low-income regions, such as parts of East Africa,^[6]^ it still indicates room for improvement. Studies from the United States^[7]^ and Europe^[8]^ have reported lower rates of poor outcomes, at approximately 4% and 5.7%, respectively, compared to 16.6% in India.^[9]^ These differences may be partly attributed to the type of cataract surgery performed. Once mastered, phacoemulsification offers distinct advantages, including smaller incisions, faster recovery, less induced astigmatism, and fewer complications. In contrast, extracapsular cataract extraction and small incision cataract surgery, still common in some low-resource settings, are associated with higher rates of postoperative complications and visual limitations.^[10]^

Several key factors in our study were found to significantly impact postoperative vision, including preoperative visual acuity, ocular comorbidities, and intraoperative complications such as zonular instability and posterior capsule rupture. Patients with preexisting ocular conditions, such as glaucoma or diabetic retinopathy, were more likely to experience suboptimal outcomes.^[11,12]^ Moreover, intraoperative complications, particularly in eyes with poor preoperative status, further compounded the risk of poor vision recovery.^[13]^ These findings are consistent with previous reports from Spain^[14]^ and Finland,^[15]^ where intraoperative events were independently associated with worse postoperative outcomes.

An important observation in our study was the unusually high prevalence of small pupils, present in 28.1% of patients, far exceeding the 6.8% reported in a large-scale cataract study.^[16]^ Small pupils are well-known risk factors for surgical difficulty and complications, including capsule rupture, vitreous loss, iris trauma, and inflammation. Additionally, the high rate of diabetes (34%) in our patient population deserves attention. Diabetes not only increases the likelihood of small pupils but also contributes to more complex ocular pathologies such as diabetic retinopathy and macular edema.^[17]^ These conditions,if undetected preoperatively or poorly managed postoperatively, can severely limit visual outcomes despite technically successful surgeries.^[18]^ While comprehensive preoperative assessment remains a critical component of cataract surgical planning, its implementation in resource-limited settings like Guyana is often constrained by limited access to diagnostic technology. Standard clinical evaluations such as fundoscopy, macular optical coherence tomography, and visual field testing should be prioritized where available, especially for patients with suspected ocular comorbidities.^[19–21]^

Although advanced imaging tools such as ultrasound biomicroscopy and anterior segment optical coherence tomography can significantly aid in detecting subtle zonular or capsular abnormalities,^[22,23]^ these modalities are not yet widely accessible in most local healthcare facilities. Nevertheless, their potential value in enhancing preoperative risk stratification should not be overlooked. As ophthalmic infrastructure in low-resource settings gradually improves, the integration of such imaging techniques—even on a selective basis—could provide important support for surgical planning, particularly in patients at higher risk of intraoperative complications such as zonular laxity or posterior capsule rupture.^[24]^

Although cataract surgery outcomes in Guyana may not match the high success rates seen in developed countries,^[25]^ the significance of these surgeries in preventing blindness is undeniable. Cataract remains the leading cause of avoidable blindness globally, and expanding access to surgery in low-resource settings is crucial for reducing the global burden of visual impairment.^[26]^ According to the World Health Organization’s *World Report on Vision*, cataract surgery is one of the most cost-effective public health interventions, capable of greatly improving quality of life and reducing the socioeconomic impact of blindness.^[1]^

Future studies should prioritize optimizing preoperative evaluations, managing comorbidities effectively during surgery, and reducing intraoperative complications. Multicenter collaborations could provide more generalizable data, enhancing the reliability of findings and identifying cost-effective strategies to improve outcomes for cataract patients in resource-limited environments.

This study has several strengths, including being the first to report postoperative visual outcomes of cataract surgery in Guyana and the use of logistic regression to identify independent risk factors. However, several limitations should be noted. The retrospective design may introduce selection bias. The follow-up period was limited to 1 month, which does not capture long-term outcomes. Additionally, the lack of advanced preoperative imaging limited our ability to fully assess subtle ocular comorbidities. These factors should be considered when interpreting our findings.

## 5. Conclusions

This study highlights ocular comorbidities and intraoperative complications, like zonular laxity and posterior capsule rupture, as key factors affecting visual outcomes after cataract surgery in Guyana. In resource-limited settings, thorough preoperative assessments and tailored postoperative care are essential for better surgical results. Future research should focus on multicenter studies and long-term follow-ups to confirm these findings and explore cost-effective ways to manage comorbidities and reduce complications.

## Author contributions

**Data curation:** Yi Liu.

**Formal analysis:** Shivannie Persaud.

**Investigation:** Yi Liu, Shailendra Sugrim.

**Methodology:** Shailendra Sugrim.

**Project administration:** Shailendra Sugrim, Carla Reid.

**Software:** Yi Liu, Carla Reid.

**Supervision:** Tiffony Persaud.

**Validation:** Shivannie Persaud, Caroline Mingo.

**Visualization:** Tiffony Persaud, Caroline Mingo.

**Writing – original draft:** Yi Liu.

**Writing – review & editing:** Yi Liu.
